# Studying the Functional Potential of Ground Ivy (*Glechoma hederacea* L.) Extract Using an In Vitro Methodology

**DOI:** 10.3390/ijms242316975

**Published:** 2023-11-30

**Authors:** Danijela Šeremet, Ksenija Durgo, Jelena Kosanović, Ana Huđek Turković, Ana Mandura Jarić, Aleksandra Vojvodić Cebin, Draženka Komes

**Affiliations:** Faculty of Food Technology and Biotechnology, University of Zagreb, Pierottijeva 6, 10 000 Zagreb, Croatia; dseremet@pbf.hr (D.Š.); kdurgo@pbf.hr (K.D.); jkosanovic@pbf.hr (J.K.); ahudek@pbf.hr (A.H.T.); amandura@pbf.hr (A.M.J.); avojvodic@pbf.hr (A.V.C.)

**Keywords:** antioxidant capacity, cellular macromolecules, cytotoxicity, ground ivy, human cell lines

## Abstract

*Glechoma hederacea* L., known as ground ivy, has a long history of use in folk medicine. The main bioactive compounds in ground ivy are polyphenolic compounds known for their potent antioxidant and antimicrobial activities and thus have high potential as functional ingredients against bacterial infections and the occurrence of chronic diseases associated with oxidative stress in the human body. The aim of the present study was to determine the biological activity of ground ivy extract on selected human cell lines, including hepatic (HepG2), tongue (CAL 27), gastric (AGS) and colon (Caco-2) cancer cell lines by evaluating cytotoxicity, formation of reactive oxygen species and genotoxicity. The antioxidant capacity of the extract was additionally evaluated using cellular model macromolecules of protein and DNA, bovine serum album and plasmid phiX174 RF1 DNA. The effect of ground ivy extract on representatives of human microflora, including *L. plantarum*, *E. coli* and *S. aureus*, was also studied. The cytotoxicity of the extract depended on the type of cells treated, and the pro-oxidant effect generally decreased with increasing exposure time. The most pronounced genoprotective effect against hydroxyl radical damage was monitored in model plasmid DNA and occurred at the highest tested concentration (0.25 mg mL^−1^), with 95.89% preservation of the supercoiled form of the plasmid. This concentration also had the most significant antioxidant activity on the model protein—14.01% more than the positive control prepared using Trolox. The ground ivy extract showed high antimicrobial potential against the pathogenic bacteria *E. coli* and *S. aureus*.

## 1. Introduction

Humans and animals have been associated with plants since the beginning of life on Earth when plants provided them with oxygen, shelter, food and medicinal preparations. As time passed and societies began to emerge, people learned to recognize and categorize plant materials that were suitable for satisfying life’s needs, and therefore, since ancient times, people have looked to nature for remedies for various diseases and ailments [1]. It is estimated that 80% of the world’s population still utilizes various plants to treat diseases, and the number is probably higher in developing countries [2]. In these countries, plants are used for therapeutic needs because they are more accessible and cheaper than synthetic drugs. Also, one-third of the population of the United States and Europe uses herbal medicines for health purposes. There are about 70,000 known plant species used to treat diseases, and only 15% have been researched for medicinal use. Despite this low rate, 25% of conventional drugs used in modern medicine are of plant origin, which leaves room for further research on the use of medicinal plants for medical purposes [3]. Although medicinal plants and their products have been used for thousands of years and scientific evidence has been accumulated for hundreds of years, it was not until the 20th century that the regulation of medicines, especially herbal medicines, began requiring data on the quality, efficacy and safety of medicines [4]. During the 1970s, exhaustive scientific studies were performed to characterize the chemical composition of plants and to introduce methods to evaluate their biological activities. In the years that followed, these studies were crucial in establishing regulations for herbal medicines in the United States and Europe [5]. The Committee on Herbal Medicinal Products, established in accordance with Regulation (EC) No 726/2004 and the Herbal Directive, is the European Medicines Agency’s committee whose obligations include compiling and assessing scientific data on herbal substances, preparations and combinations to support the harmonization of the European market.

The *Lamiaceae* family is one of the largest botanical families, with a high number of plants possessing various biological activities, and has thus found application in medicinal practices. The *Lamiaceae* family is well-known for a variety of aromatic spices such as oregano, thyme, sage, savory, mint, basil, rosemary, lemon balm, etc. [6]. The underutilized potential of the *Lamiaceae* family includes plants with a long tradition of use in folk medicine but insufficiently researched chemical and bioactive composition such as ground ivy (*Glechoma hederacea* L.). Ground ivy has been used for generations as a traditional herbal preparation—dried leaves in infusions to treat respiratory diseases such as asthma, bronchitis, colds, coughs and sore throats, drinks made from the fresh plant for digestive problems and decoctions externally to treat skin irritations. In addition, ground ivy has also been used as a diuretic and stimulant, as well as for the treatment of headaches, arthritis, scurvy, jaundice, menstrual or puerperal disorders and mental disorders such as hypochondria and monomania. The aforementioned biological activities are associated with the antioxidant capacity of ground ivy due to the presence of various bioactive compounds, especially polyphenolic compounds [7]. The most represented phenolic compounds in ground ivy are phenolic acids such as rosmarinic, protocatechuic, caffeic, chlorogenic, cryptochlorogenic and ferulic acids, while among the flavonoids, the presence of rutin, isoquercetin, genistin, genistein and daidzin has been reported [8,9,10]. Based on the studies conducted so far, ground ivy has pronounced pharmacological properties. In the study by Kumarasamy et al. [11], methanolic extract of ground ivy showed an inhibitory effect against 14 bacterial species. The anti-inflammatory and antioxidant potential of ground ivy water extract was demonstrated in an in vitro study by Chou et al. [10], where it prevented lipopolysaccharide-induced DNA damage in RAW264.7 macrophages, reduced the content of lipopolysaccharide-induced nitric oxide and malondialdehyde, increased glutathione levels and regulated antioxidant enzyme activities. Further, in an in vivo study conducted using rat models of cholestatic liver injury, ground ivy extract showed protective effects against cholestatic liver injury, as evidenced by the improvement in serum biochemicals, ductular reaction, oxidative stress, inflammation and fibrosis [12]. The anticancer properties of ground ivy water extract were demonstrated in synergy with cisplatin in human renal cancer cell lines (786-O). Cell cycle analysis showed that the combination of ground ivy extract and cisplatin exerted cytotoxicity by inducing G2/M arrest and apoptosis [13]. A study by Qiao et al. [14] investigated the effect of ground ivy extract on melanogenesis in B16 melanoma cells. The results showed inhibition of melanin synthesis in B16 melanoma cells due to the inhibition of tyrosinase gene transcription. Considering that cytotoxicity against melanoma cells was not proven but depigmentation was, the ground ivy extract shows potential for use in the prevention of hyperpigmentation [14].

The aim of the present study was to determine the biological activity of ground ivy extract on selected human cell lines, including hepatic (HepG2), tongue (CAL 27), gastric (AGS) and colon (Caco-2) cancer cell lines, by evaluating cytotoxicity, formation of reactive oxygen species and genotoxicity at concentrations that are similar to the ones consumed daily. The antioxidant capacity of the extract was additionally evaluated using cellular model macromolecules of protein and DNA, bovine serum album and plasmid phiX174 RF1 DNA. The effect of ground ivy extract on representatives of human microflora, including *Lactobacillus plantarum*, *Escherichia coli* and *Staphylococcus aureus*, was also studied. The obtained results will be further utilized in the determination of the concentration of ground ivy extract that can be safely used in the formulation of functional foods, simultaneously providing desirable antioxidative properties.

## 2. Results and Discussion

### 2.1. Determination of Cytotoxic/Proliferative Effects of Ground Ivy Extract

#### 2.1.1. Cytotoxic/Proliferative Effects of Ground Ivy Extract on Continuous Human Cell Lines

**Figure 1:**
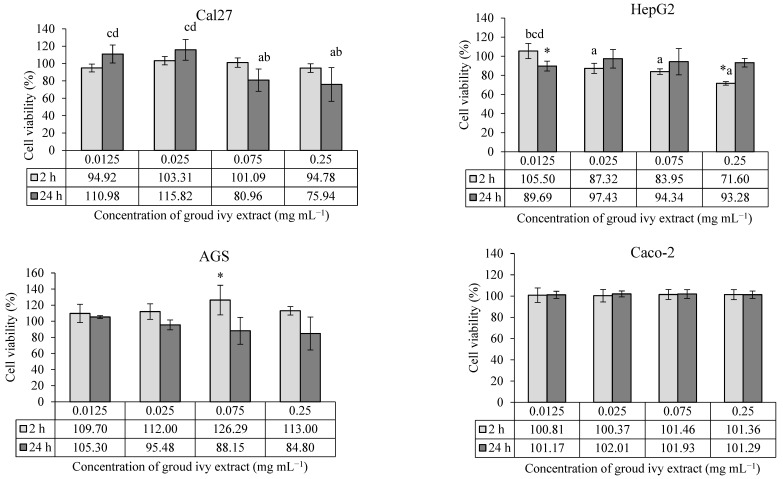
Cytotoxic/proliferative effects of ground ivy extract on continuous human cell lines. *, statistically significant difference compared with the control (*p* < 0.05); a, statistically significant difference compared with 0.0125 mg mL^−1^ (*p* < 0.05); b, statistically significant difference compared with 0.025 mg mL^−1^ (*p* < 0.05); c, statistically significant difference compared with 0.075 mg mL^−1^ (*p* < 0.05); d, statistically significant difference compared with 0.25 mg mL^−1^ (*p* < 0.05).

The cytotoxic/proliferative effects of ground ivy extract on the human continuous cell lines Cal27, HepG2, AGS and Caco-2 are shown in Figure 1.

According to the presented results, the cytotoxic effect of ground ivy extract depends on the type of cell line and the exposure time. In Cal27 tongue epithelial cells, none of the tested concentrations showed a statistically significant effect (*p* > 0.05) on cell survival compared with the control. However, a statistically significant (*p* < 0.05) decrease in cell survival was observed at higher concentrations (0.075 and 0.25 mg mL^−1^) compared with lower concentrations (0.0125 and 0.025 mg mL^−1^) after 24 h of treatment, indicating the cytotoxic effect of the extract. The cytotoxic effect of ground ivy extract may be associated with the antiproliferative properties of rosmarinic acid, the most abundant polyphenolic compound in the extract. Luo et al. [15] reported that rosmarinic acid proportionally inhibited the proliferation of the oral cancer cell line SCC-15 with increasing concentration (2.5–160 μM) after 24 h of treatment. The mechanism of the anticancer effect of rosmarinic acid is based on the induction of apoptotic proteins, leading to the programmed death of oral cancer cells. On the other hand, Chou et al. [13] investigated the effect of ground ivy extract and rosmarinic acid on the human renal proximal tubule epithelial cell line (HK-2) and human renal carcinoma cell line (RCC 786-O). The results of their research showed that the extract and rosmarinic acid significantly reduced the proliferation of the RCC 786-O tumor cell line (<50% survival) only at the highest tested concentration of 800 μg mL^−1^, while no toxic effect was detected in the HK-2 cells during the 24 h of treatment. In addition, their studies reported that the extract and rosmarinic acid, when combined with the conventional chemotherapeutic agent cisplatin, enhanced antiproliferative effects against RCC 786-O tumor cells while attenuating the side effects in normal HK-2 cells [13]. By comparing the results of previous studies with the results of the present study, it can be concluded that the sensitivity of cells to the effect of the extract depends on the type of cells; thus, although all the cells used are epithelial cells, they have different specific processes that allow them to adapt to a certain level of new conditions depending on the type of organ they cover.

In the case of HepG2 liver cells, treatment with the lowest concentration (0.0125 mg mL^−1^) of the extract for 24 h and the highest concentration (0.25 mg mL^−1^) for 2 h showed statistically significant (*p* < 0.05) decreases in cell survival compared with the control. In addition, a statistically significant (*p* < 0.05) dose–dependent cytotoxic effect was observed at the higher concentrations (0.025, 0.075 and 0.25 mg mL^−1^) compared with the lowest concentrations during the 2 h treatment. By extending the incubation period for HepG2 cells to 24 h, an increase in cell viability was observed, regardless of concentration. The recovery of HepG2 cells after 24 h of treatment may be associated with the presence of enzyme systems for the detoxification of xenobiotics and the high regenerative capacity of the cells. There is also a possibility that the metabolites produced during the degradation of rosmarinic acid are less cytotoxic, which consequently leads to the stimulation of cell growth. However, Chao et al. [16] reported that the treatment of HepG2 cells with the ethyl acetate fraction of ground ivy extract for 24 h resulted in a concentration-dependent decrease in the growth of HepG2 cells, with an IC_50_ value of 350 μg mL^−1^. One possible reason for the discrepancy between the results of the aforementioned and present studies is the difference in the solvent used for extraction. Organic solvents extract more hydrophobic components, which are often responsible for the antiproliferative activity, from plants compared with water.

In the case of AGS cells, 0.075 mg mL^−1^ of the ground ivy extract was the only concentration that had a statistically significant increase (*p <* 0.05) in the survival rate compared with the control after treatment for 2 h, while in the case of Caco-2 cells, the extract had no effect on survival rate at the tested concentrations, regardless of the treatment duration (2 h or 24 h). Grabowska et al. [8] also reported no cytotoxic effect of water or ethanol extracts of ground ivy on Caco-2 cells at the tested concentrations (10–100 μg mL^−1^).

#### 2.1.2. Cytotoxic/Proliferative Effects of Ground Ivy Extract on *L. plantarum*, *E. coli* and *S. aureus*

**Figure 2:**
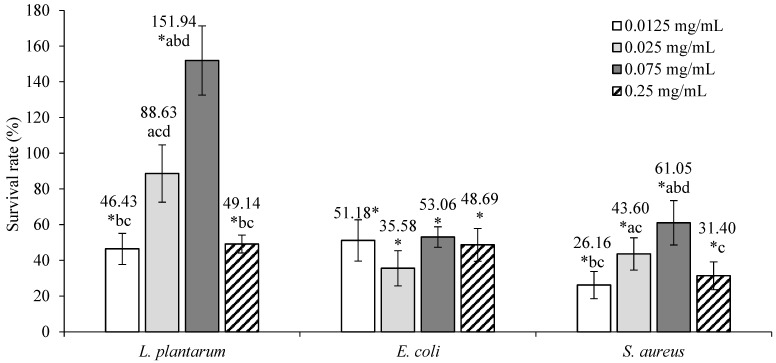
Cytotoxic/proliferative effects of ground ivy extract on representatives of human microflora. *, statistically significant difference compared with the control (*p* < 0.05); a, statistically significant difference compared with 0.0125 mg mL^−1^ (*p* < 0.05); b, statistically significant difference compared with 0.025 mg mL^−1^ (*p* < 0.05); c, statistically significant difference compared with 0.075 mg mL^−1^ (*p* < 0.05); d, statistically significant difference compared with 0.25 mg mL^−1^ (*p* < 0.05).

The cytotoxic/proliferative effects of ground ivy extract on *L. plantarum*, *E. coli*, and *S. aureus* are shown in Figure 2. 

Ground ivy extracts of 0.0125 and 0.25 mg mL^−1^ concentrations resulted in statistically significant decreases (*p* < 0.05) in the viability of *L. plantarum* compared with the control. A 0.025 mg mL^−1^ concentration of the extract had no statistically significant effect (*p >* 0.05) on the survivability of the bacteria, while a significant increase (*p* < 0.05) in survivability was observed at a concentration of 0.075 mg mL^−1^. This effect can be explained by the fact that the ground ivy extract prepared with water as a solvent contains numerous water-soluble polysaccharides that have prebiotic properties and thus stimulate the growth and proliferation of bacteria. The content of soluble dietary fiber in the ground ivy was found to be in the range of ~5–10% of the dry matter [9].

The survival of *E. coli* and *S. aureus* was significantly reduced (*p* < 0.05) at all tested concentrations compared with the control, from which it follows that the extract has a bacteriostatic effect against both pathogenic microorganisms. For *E. coli*, no statistically significant differences (*p* > 0.05) were observed between the tested extract concentrations, implying that the extract has the same bacteriostatic effect at all concentration ranges; for *S. aureus*, the most pronounced bacteriostatic effects were observed at concentrations of 0.0125 mg mL^−1^ and 0.25 mg mL^−1^, which statistically showed the same bacteriostatic activity (*p* > 0.05). In the study by Kumarasamy et al. [11], the methanolic extract of ground ivy showed inhibitory activity against 14 bacterial species, including *E. coli* and *S. aureus,* with minimum inhibitory concentrations of 5.00 and 2.5 × 10^−1^ mg mL^−1^, while in the study by Coss et al. [17], a water–ethanol extract of ground ivy showed very low antimicrobial activity against *E. coli* and *S. aureus*. The same authors [17] reported that this does not necessarily mean that ground ivy does not contain significant amounts of bioactive compounds responsible for antimicrobial activity and that it may act in an indirect manner, meaning it may contain precursor molecules that need to be bioactivated in the body. Gwiazdowska et al. [18] studied the antimicrobial activity of ground ivy extract prepared via supercritical CO_2_ extraction and found the strongest antimicrobial activity against *S. aureus* with a minimum inhibitory concentration of 0.3 mg mL^−1^ and noted a higher sensitivity of Gram-positive bacteria compared with Gram-negative bacteria.

It is known that polyphenolic compounds contribute to the modulation of the composition of the intestinal microbiota reaching the colon and that the intestinal microbiota can convert them into metabolites with prebiotic effects. Certain doses of polyphenolic compounds can inhibit various groups of pathogenic bacteria while promoting the development of probiotic microorganisms [19]. In pathogenic microorganisms, polyphenolic compounds primarily lead to the disruption of cell membrane integrity and the disruption of the electron respiratory chain, resulting in reduced or complete absence of microorganism growth and consequent loss of virulence. Auto-oxidizing polyphenols can also act as pro-oxidants, with oxidation of polyphenols producing hydrogen peroxide, which induces the formation of breaks in DNA and thus has a bactericidal effect [20]. The cytotoxic effect of ground ivy extract against *E. coli* and *S. aureus* can be associated with the antimicrobial properties of rosmarinic acid, which have been demonstrated in previous studies. Abedini et al. [21] reported that rosmarinic acid and its ester methyl rosmarinate are the main antimicrobial components of the water–methanol extract of *Hyptis atrorubens* from the *Lamiaceae* family, inhibiting the growth of 29 of the 46 microbial species tested, including *S. aureus*. Based on available data, Gram-negative bacteria generally had higher resistance to antimicrobial compounds compared with Gram-positive bacteria. For example, in the study by Alagawany et al. [22], rosemary extract, which is rich in rosmarinic acid, had a strong inhibitory effect on the growth of Gram-positive bacteria but was not effective against Gram-negative bacteria, including *E. coli* and *S. typhimurium* [22]. A similar trend has been observed with other extracts of plants from the *Lamiaceae* family, such as thyme extract in the study by Generalić Mekinić et al. [23]. On the other hand, Sage extract from the same study [23] not only successfully acted as a bacteriostat against Gram-positive bacteria but also showed inhibitory activity against the Gram-negative bacteria *E. coli* and *S. infantis*, albeit at much higher doses. The probable reason for this is the presence of an outer membrane that envelops the cell wall and thus provides greater protection for Gram-negative bacteria [23]. A parameter that also plays an important role in the antibacterial activity of plant extracts is the solvent used for the extraction of bioactive compounds from the plant material. In the study by Alagawany et al. [22], a methanol extract of rosemary containing 30% carnosic acid, 16% carnosol and 5% rosmarinic acid acted as a more effective antimicrobial agent against a wider range of microorganisms compared with aqueous extract of rosemary containing 15% rosmarinic acid. Therefore, it can be concluded that the antimicrobial potential of rosmarinic acid in plant extracts is enhanced in synergy with the mechanisms of action of other bioactive compounds and varies depending on the presence of other compounds. On the other hand, the stimulatory effect of a 0.075 mg mL^−1^ concentration of ground ivy extract on the growth of *L. plantarum* may be associated with the demonstrated role of rosmarinic acid in improving the functions of the gut microbiota, as probiotic bacteria can metabolize polyphenolic compounds [22]. Therefore, based on previous research and the results of the present study, bioactive compounds in ground ivy extract possess potent antimicrobial activity against the pathogenic bacteria *E. coli* and *S. aureus*. This is why the extract has the potential for use as a natural preservative in the food industry, while its use as a prebiotic is questionable due to the opposite effect with very small differences in the applied doses, which in case of an inhibitory effect on probiotic bacteria may lead to disruption of the human microflora and, consequently, to disorders of the digestive tract and the immune system.

### 2.2. Determination of Antioxidative/Pro-Oxidative Effects of Ground Ivy Extract 

#### 2.2.1. Antioxidative/Pro-Oxidative Effects of Ground Ivy Extract on Continuous Human Cell Lines

**Figure 3:**
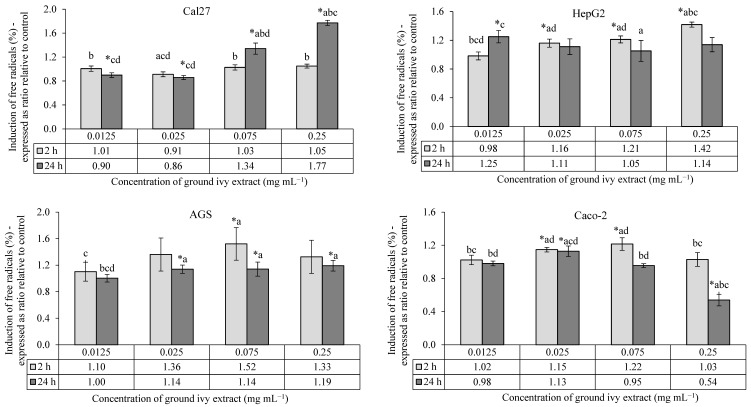
Antioxidant/pro-oxidant effects of ground ivy extract on continuous human cell lines. *, statistically significant difference compared with the control (*p* < 0.05); a, statistically significant difference compared with 0.0125 mg mL^−1^ (*p* < 0.05); b, statistically significant difference compared with 0.025 mg mL^−1^ (*p* < 0.05); c, statistically significant difference compared with 0.075 mg mL^−1^ (*p* < 0.05); d, statistically significant difference compared with 0.25 mg mL^−1^ (*p* < 0.05).

The antioxidant/pro-oxidant effects of ground ivy extract on the continuous human cell lines Cal27, HepG2, AGS and Caco-2 are shown in Figure 3.

All tested concentrations of ground ivy extract showed no statistically significant (*p* > 0.05) effects against the induction of free radicals in Cal27 cells after 2 h of treatment compared with the control. After 24 h of treatment, lower concentrations (0.0125 and 0.025 mg mL^−1^) showed antioxidant activity, while higher concentrations (0.075 and 0.25 mg mL^−1^) showed pro-oxidant activity. The extract had a dose–response effect, with an increase in the concentration of free radicals after 24 h.

Compared with the control, a significant (*p* < 0.05) increase in the concentration of free radicals was observed in HepG2 cells after 2 h of treatment with all except the lowest concentrations of extract (0.0125 mg mL^−1^); however, after 24 h of treatment, there was no statistically significant difference in comparison to the control. Liver HepG2 cells are metabolically active, thus this result is expected. The presence of free radicals was monitored after initial exposure (2 h of treatment) to a mixture of compounds that are metabolized in the liver cells, but their presence returned to levels similar to those in untreated cells over the next 24 h. Additional damage to cellular macromolecules caused by free radicals in liver cells is not expected with prolonged exposure to ground ivy extract. 

In AGS cells, a statistically significant pro-oxidant effect (*p* < 0.05) was observed after 2 h of treatment only at a concentration of 0.075 mg mL^−1^, while a significant dose–response pro-oxidant effect (*p* < 0.05) was observed after 24 h of treatment at all concentrations except the lowest (0.0125 mg mL^−1^). 

After 2 h of treatment of Caco-2 cells, significant pro-oxidant activity (*p* < 0.05) was observed at concentrations of 0.025 and 0.075 mg mL^−1^, while after 24 h, pro-oxidant activity was observed only at the 0.025 mg mL^−1^ concentration, with antioxidant activity being observed at the highest tested concentration (0.25 mg mL^−1^).

Based on the obtained results, it can be concluded that the antioxidant potential of ground ivy extract varies depending on the cell type, same as was noted for the cytotoxic activity. When HepG2, AGS and Caco-2 cells were exposed to the extract for 24 h, the pro-oxidant effect decreased compared to when the cells were exposed to a shorter treatment duration; however, it increased in Cal27 cells at higher concentrations. The extract showed the strongest antioxidant activity at the highest tested concentration (0.25 mg mL^−1^) during 24 h of treatment of Caco-2 cells, while the same concentration of extract significantly stimulated free radical production in Cal27 cells over a longer exposure period. The production of free radicals was directly associated with a decrease in the viability of Cal27 and HepG2 cells, whereas this correlation was not observed in AGS and Caco-2 cells.

Excessive production of ROS in cells leads to changes in redox potential that can result in oxidative damage to cellular macromolecules. Because oxidative stress has been linked to cancer and other chronic diseases, scientific research has focused primarily on the antioxidant properties of plant polyphenols. However, these potent antioxidants may also have a pro-oxidant characteristic that depends largely on pH, the presence of transition metals, chelating ability and solubility. The instability of polyphenols in an alkaline medium leads to their autoxidation and the increased production of ROS [24].

In HepG2 and Cal27 cells, the formation of free radicals was directly associated with cell survival from which it follows that ROS, which are formed by the auto-oxidation of bioactive compounds in the ground ivy extract, play an important role in damaging cellular macromolecules and inducing cell death, i.e., they are responsible for the cytotoxic effect of the extract. On the other hand, the induction of free radicals in AGS cells was dependent on the concentration of the extract, i.e., as the extract concentration increased, the formation of free radicals also increased. However, the free radical concentration was higher during the 24 h of treatment than after 2 h. It follows that free radicals might induce antioxidant mechanisms in cells, but this capacity is weakened with increasing concentration of the extract, which was reflected in the survival of AGS cells after prolonged treatment, i.e., the pro-oxidant character of the ground ivy extract increased with increasing concentration. The obtained results can be explained by the fact that cancer cells have increased concentrations of ROS compared with normal cells but are more sensitive to disturbances in the redox balance, which can activate various signaling pathways leading to cell death [25].

To determine the effect of biologically active compounds in ground ivy extract on cellular macromolecules, its antioxidative/pro-oxidative effects on model cellular macromolecules were investigated.

#### 2.2.2. Antioxidative/Pro-Oxidative Effects of Ground Ivy Extract on a Model Cellular Protein: Bovine Serum Albumin 

**Figure 4:**
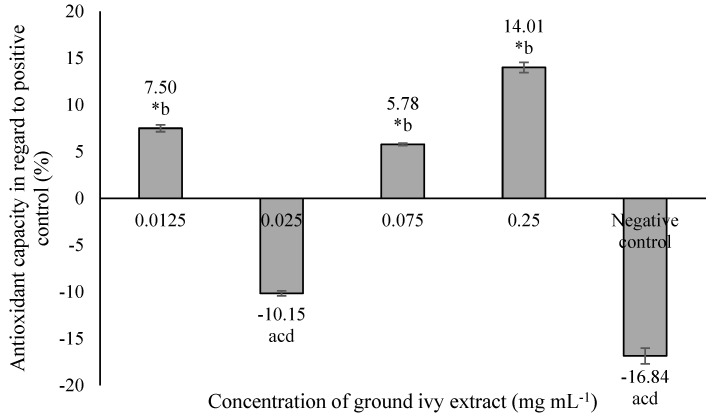
Effect of ground ivy extract on protein (bovine serum albumin) carbonylation. *, statistically significant difference compared with the negative control (*p* < 0.05); a, statistically significant difference compared with 0.0125 mg mL^−1^ (*p* < 0.05); b, statistically significant difference compared with 0.025 mg mL^−1^ (*p* < 0.05); c, statistically significant difference compared with 0.075 mg mL^−1^ (*p* < 0.05); d, statistically significant difference compared with 0.25 mg mL^−1^ (*p* < 0.05).

The antioxidant capacity of ground ivy extract on a model cellular protein, bovine serum albumin, is shown in Figure 4.

The most significant antioxidant activity—14.01% more than the positive control—was obtained at the highest tested concentration of 0.25 mg mL^−1^, while the 0.025 mg mL^−1^ concentration resulted in a pro-oxidant effect that was statistically equal (*p >* 0.05) to that of the negative control. The antioxidant capacity of ground ivy extract can be attributed to the presence of polyphenolic compounds; this is supported by the results of the research by Ou et al. [26], who reported the inhibitory effect of rosmarinic acid on the formation of protein carbonyls in the BSA protein model with increasing concentration (6.25–400 μg mL^−1^).

#### 2.2.3. Antioxidative/Pro-Oxidative Effects of Ground Ivy Extract on a Model DNA: Plasmid phiX174 RF1 DNA

**Figure 5:**
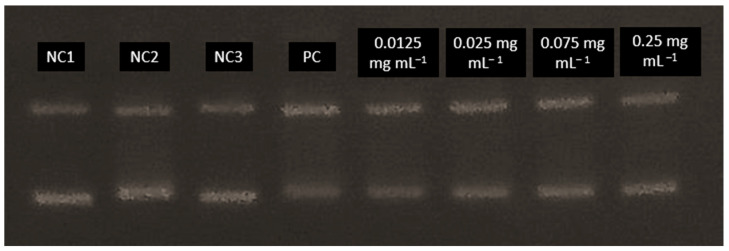
Gel stained with ethidium bromide and irradiated with UV radiation. NC, negative controls; PC, positive control.

The potential genoprotective effect of ground ivy extract was determined using the plasmid phiX174 RF1 DNA. In addition to the effect of the extract, the plasmid was exposed to hydroxyl radicals generated by the action of UV radiation on H_2_O_2_. After treatment, agarose electrophoresis was performed, and the resulting bands were visualized by staining the gel with ethidium bromide solution (Figure 5). When DNA damage occurs, the plasmid changes its conformation from a supercoiled form to a relaxed form, resulting in its slower movement through the gel.

**Figure 6:**
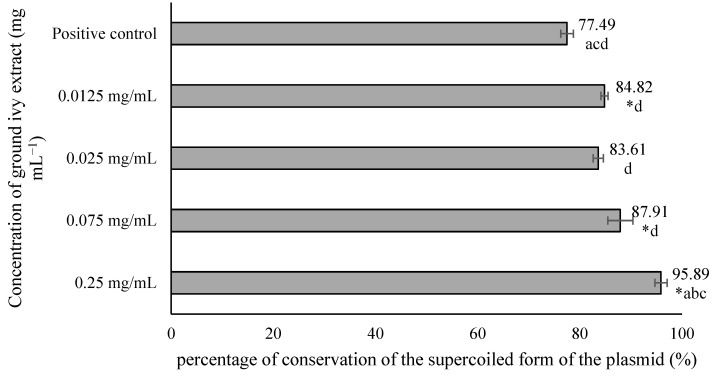
Effect of ground ivy extract on plasmid phiX174 RF1 DNA. *, statistically significant difference compared with the positive control (*p* < 0.05); a, statistically significant difference compared with 0.0125 mg mL^−1^ (*p* < 0.05); b, statistically significant difference compared with 0.025 mg mL^−1^ (*p* < 0.05); c, statistically significant difference compared with 0.075 mg mL^−1^ (*p* < 0.05); d, statistically significant difference compared with 0.25 mg mL^−1^ (*p* < 0.05).

From the results shown in Figure 5, it is evident that in the negative controls—NC1, NC2 and NC3—most of the plasmids were in the supercoiled conformation, which was reflected in the significantly stronger band intensity of the supercoiled plasmid compared with the relaxed plasmid. In contrast, in the positive control PC4, the relaxed form of the plasmid is more pronounced, providing a stronger signal compared with the supercoiled conformation. Based on the obtained results, it can be concluded that exposure of the plasmid to UV radiation only or to H_2_O_2_ only does not lead to significant DNA damage in contrast to the synergistic effect of these two agents, which results in a much stronger signal of the relaxed form. Therefore, the mean value of the band intensities of 3 negative controls was taken and considered as one value representing 100% conservation of the supercoiled form of the plasmid. The results are shown in Figure 6. 

All tested concentrations of ground ivy extract, except 0.025 mg mL^−1^, provided significant (*p* < 0.05) protection against damage to genetic material compared with the positive control. The most pronounced genoprotective effect was obtained at the highest tested concentration (0.25 mg mL^−1^), with 95.89% preservation of the supercoiled form of the plasmid. Oalđe et al. [27] studied the effect of methanolic, ethanolic and aqueous ground ivy extracts on the protection of plasmid supercoiled DNA (pUC19) against the hydroxyl radical. The same authors [27] reported that extracts showed concentration-dependent genoprotective activity against hydroxyl radicals, with a 1 mg mL^−1^ concentration of aqueous extracts providing the best DNA protection, with ~10% of the open form of plasmid DNA recorded. For comparison, for the positive control (UV-treated plasmid without sample treatment), ~75% of the open form of plasmid DNA was recorded. The same authors also stated that potent genoprotective activity is not due only to the presence of phenolic compounds, but also to other non-phenolic compounds such as carbohydrates and terpenes, which are also found in the water extracts [27].

### 2.3. Determination of Genotoxic Activity of Ground Ivy Extract on Continuous Human Cell Lines

**Figure 7:**
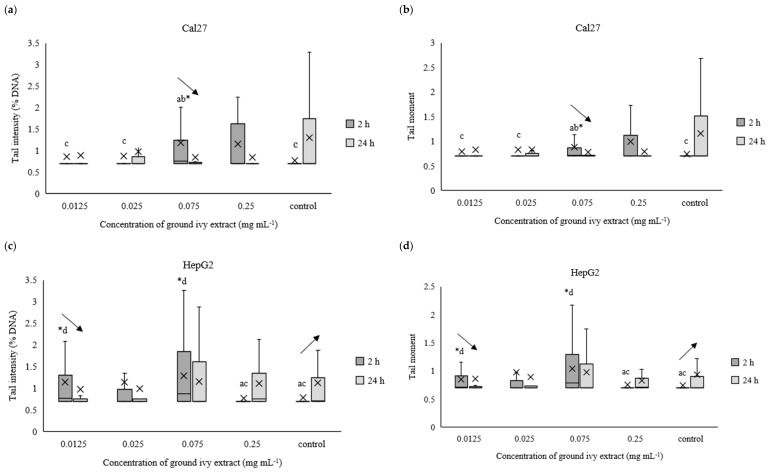

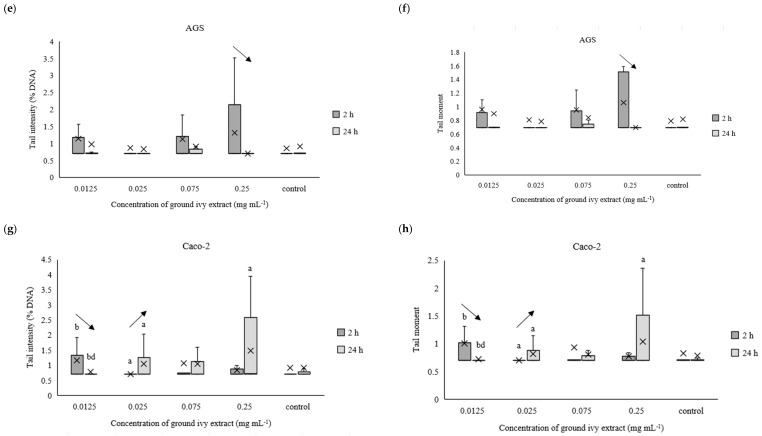
Effect of ground ivy extract on genetic materials of Cal27 (**a**,**b**), HepG2 (**c**,**d**), AGS (**e**,**f**) and Caco-2 (**g**,**h**) cells determined by tail intensity (**a**–**d**) and tail moment. *, statistically significant difference compared with the control (*p* < 0.05); a, statistically significant difference compared with 0.0125 mg mL^−1^ (*p* < 0.05); b, statistically significant difference compared with 0.025 mg mL^−1^ (*p* < 0.05); c, statistically significant difference compared with 0.075 mg mL^−1^ (*p* < 0.05); d, statistically significant difference compared with 0.25 mg mL^−1^ (*p* < 0.05); -, statistically significant difference (increase or decrease depending on the direction of the arrow) between 2 and 24 h treatments at the same concentration.

The potential genotoxic effect of ground ivy extract on DNA in the continuous human cell lines Cal27, HepG2, AGS and Caco-2 was measured using the comet assay after exposures for 2 h or 24 h. For easier processing of the obtained results, the data were normalized to the natural logarithm. The results are presented in Figure 7a–h.

After 2 h of treating Cal27 cells, only the 0.075 mg mL^−1^ concentration caused a significant (*p* < 0.05) increase in tail intensity and tail moment compared with the control, whereas the same parameters did not change significantly (*p* > 0.05) in the entire concentration range after prolonged treatment (24 h) of the Cal27 cells (Figure 7a,b). Regarding the HepG2 cells, the 0.075 mg mL^−1^ concentration also caused a significant (*p* < 0.05) increase in tail intensity and tail moment compared with the control after 2 h of treatment. Additionally, damage to the genetic material was also observed at the lowest tested concentration of 0.0125 mg mL^−1^. Further, no significant (*p* > 0.05) genoprotective nor genotoxic effect of ground ivy extract was observed in the AGS cells (Figure 7e,f), regardless of treatment time. Similar results were obtained for the Caco-2 cells—no significant (*p* > 0.05) changes in the genetic material were observed compared with the control, but significant decreases (*p* < 0.05) in both tail intensity and tail moment were observed when the concentration of the ground ivy extract was increased from 0.0125 to 0.025 mg mL^−1^. Comparing the results with those of the antioxidant effect on the model DNA (Section 2.2.3.), where the protective effect increased with the increase in extract concentration, showed that this dependence was not observed when the same effect was studied on cell lines.

Decreases in tail intensity and tail moment with increases in incubation times for Cal27, HepG2, AGS and Caco-2 cells at concentrations of 0.075, 0.0125, 0.25 and 0.0125 mg mL^−1^, respectively, were observed. This observation may suggest that the active compounds in ground ivy extract not only protect against damage to genetic material but also participate in DNA repair. The potential genoprotective activity may be partly due to the presence of rosmarinic acid in the ground ivy extract. In an in vitro study of the genoprotective effect of polyphenolic compounds on the neuronal PC12 cell model, Silva et al. [28] showed that rosmarinic acid was involved in the repair of oxidized bases as well as breaks in DNA, thus protecting genetic material from oxidative damage. Relating the obtained results of the genoprotective effect to cell viability, it can be assumed that the cytotoxic effect of the extract results from damage to protein or lipid components of membranes and not genetic material. However, further studies are needed to confirm this.

## 3. Materials and Methods

### 3.1. Materials

A voucher for the collected ground ivy (April 2020; Bjelovar, Croatia) is stored in the Flora Croatica Database (University of Zagreb, Faculty of Science, Department of Botany, Croatia) under label 71767.

For the experiment, the roots of the collected ground ivy were separated from the aerial parts. The aerial parts were air-dried until the dry matter content was >90%. They were then ground and sieved, and the fraction below 450 µm was used for the preparation of the extract.

### 3.2. Biological Test Systems

Hepatocellular carcinoma cells (HepG2), tongue epithelial carcinoma cell line (CAL 27), gastric carcinoma (AGS) and adenocarcinoma colon cells (Caco-2) were provided by ECACC (European Collection of Authenticated Cell Cultures, UK). *Escherichia coli*, *Lactobacillus plantarum* and *Staphylococcus aureus* are part of the collection of the Laboratory for Biology and Microbial Genetics (Faculty of Food Technology and Biotechnology, University of Zagreb, Zagreb, Croatia).

### 3.3. Chemicals

Rosmarinic acid (97%), caffeic acid (HPLC standard), cryptochlorogenic acid (>98%), chlorogenic acid (95%), rutin trihydrate (>97%), neutral red (NR), 2,4-dinitrophenylhydrazine (DNPH), dichlorofluorescein diacetate (DCF-DA), low melting point agarose, normal melting point agarose, EDTA disodium salt, bovine serum albumin, ethidium bromide, guanidine hydrochloride, trichloroacetic acid and (S)-6-Methoxy-2,5,7,8-tetramethylchromane-2-carboxylic acid (Trolox) were purchased from Sigma-Aldrich (St. Louis, MO, USA). Formic acid was purchased from Carlo Erba (Emmendingen, Germany) and acetonitrile from Fisher Scientific (Waltham, MA, USA). Hydrogen peroxide, glycerol, iron(III) chloride, ascorbic acid, bromothymol blue and glacial acetic acid were supplied by Kemika (Zagreb, Croatia). Methanol was supplied by Panreac (Barcelona, Spain). Ethyl acetate was purchased from Lach-Ner (Neratovice, Czech Republic) and Tris-HCl from Invitrogen (SAD). Plasmid phiX174 RF1 DNA was supplied by Promega (Fitchburg, WI, USA). 

### 3.4. Methods

#### 3.4.1. Preparation of Ground Ivy Extract

Extraction was performed using 1 g of sample and 100 mL of demineralized water for 10 min in a 100 °C water bath (Inko VKZ ERN, Inkolab d.o.o., Zagreb, Croatia). After completion of the extraction, the extract was centrifugated (Thermo Scientific SL8/8R centrifuge, Waltham, MA, USA) at 9500 rpm for 20 min and 4 °C and the supernatant was concentrated to a 10-fold volume under vacuum (IKA RV8, Staufen, Germany) and subjected to freeze drying (Alpha 1-2 LD plus freeze-dryer, Martin Christ, Osterode am Harz, Germany) to obtain a lyophilizate of the extract (referred to as ‘extract’ in the text), which was used for further experiments. 

#### 3.4.2. Characterization of the Phenolic Profile of Ground Ivy Extract

HPLC analysis was performed on the Agilent Series 1200 chromatographic system (Agilent Technologies, Santa Clara, CA, USA) coupled with a photodiode array detector and a Zorbax Extend C18 (4.6 × 250 mm, i.d., 5 μm) chromatographic column (Agilent Technologies, Santa Clara, CA, USA). The elution was performed in a gradient with a two-component mobile phase consisting of 1% (*v*/*v*) formic acid solution in water and 1% (*v*/*v*) formic acid solution in acetonitrile as described in the study by Šeremet et al. [9]. The results are presented in Table 1.

#### 3.4.3. Determination of Cytotoxicity against Human Cancer Cell Lines

The cytotoxicity of ground ivy extract was determined using a neutral red (NR) assay, as described previously [29]. Briefly, 100 μL of 10^5^ cells mL^−1^ was inoculated into the microtiter plates. After 24 h of cultivation, the cells were treated with 100 μL solutions of different concentrations of the ground ivy extract (0.0125, 0.025, 0.075 and 0.25 mg mL^−1^) previously prepared in the appropriate culture medium. The negative control contained 100 μL of the appropriate culture medium. The treatment of the cells lasted for 2 h or 24 h. At the end of the treatment, cells were washed and 100 μL of the NR solution was added. After incubation at 37 °C for 45 min, cells were washed and accumulated neutral red was extracted using a destaining solution (distilled water:ethanol:glacial acetic acid = 1:0.98:0.02, *v*/*v*/*v*). The absorbance was measured at 540 nm in a microtiter reader (Cecil Instruments Ltd., Cambridge, UK). Cell viability was calculated using Equation (1):

(1) $$cell\;viability\;\left(\%\right)=\frac{{Absorbance}_{sample}}{{Absorbance}_{control}}\times 100$$

#### 3.4.4. Determination of Cytotoxicity against Representatives of Human Microflora

Bacterial cultures (*Escherichia coli*, *Lactobacillus plantarum* and *Staphylococcus aureus*) were grown to the exponential growth phase in appropriate liquid culture media. After washing, 100 μL of the bacterial suspension was inoculated into a 96-well microtiter plate. The bacterial suspension was treated with 100 μL of different concentrations (0.0125, 0.025, 0.075 and 0.25 mg mL^−1^) of the ground ivy extract. A bacterial suspension treated with 100 μL of the appropriate liquid culture medium served as a negative control. After 40 min of incubation at 37 °C, microdilutions were prepared and subsequently, 10 μL of each dilution was inoculated onto the appropriate solid culture media. The cells were grown at 37 °C for 24 h. The number of colonies grown (CFUs—colony-forming units) was then counted. The survival rates of bacteria after treatment with different concentrations of ground ivy extract were calculated according to Equation (2):

(2) $$Survival\;rate\;\left(\%\right)=\frac{\frac{CFU}{mL}\;bacterial\;suspension}{\frac{CFU}{mL}\;control}\times 100$$

#### 3.4.5. Determination of Reactive Oxygen Species in Human Cell Lines

The formation of reactive oxygen species (ROS) after treatment with ground ivy extract was determined using dichlorohydrofluorescein (DCF) as described previously [30,31]. A 100 μL suspension of cells with an initial concentration of 10^5^ cells mL^−1^ was inoculated into black microtiter plates. The cells were cultured for 24 h in an incubator with a controlled atmosphere (5% CO_2_) at a temperature of 37 °C. After the formation of a subconfluent monolayer, cells were washed and treated with 100 μL solutions of different concentrations of ground ivy extract (0.0125, 0.025, 0.075 and 0.25 mg mL^−1^). The negative control contained 100 μL of the appropriate culture medium. Treatment of the cells lasted for 2 h or 24 h. After treatment, the growth medium was dispensed, cells were washed and 100 μL of DCF-DA solution was added to each well. After incubation at 37 °C for 30 min, the fluorescence was measured (Cecil Instruments Ltd., Cambridge, UK) at an excitation wavelength of 485 nm and an emission wavelength of 520 nm. The measured fluorescence was compared with the fluorescence of the control and the results were expressed as a percentage of ROS induction according to Equation (3):

(3) $$Induction\;of\;free\;radicals\;\left(\%\right)=\frac{\frac{{Fluorescence}_{sample}}{Cell\;survival}}{\frac{{Fluorescence}_{control}}{100}}$$

#### 3.4.6. Determination of Antioxidant Effect on Model Protein Macromolecules

Bovine serum albumin (BSA) was used as a model cellular macromolecule to test the effect of ground ivy extract on protein carbonylation. The reaction mixture contained 240 μL BSA (1 mg mL^−1^), 60 μL iron(III) chloride solution, 60 μL ascorbic acid, 24 μL hydrogen peroxide solution (25 mM), corresponding volumes of ground ivy extract with final concentrations of 0.0125, 0.025, 0.075 and 0.25 mg mL^−1^ and distilled water to a final volume of 600 μL. The positive control contained 125 μL Trolox (10 mg mL^−1^) instead of the extract, while the negative control contained 125 μL methanol. Samples were incubated at 37 °C for 30 min. After incubation, 500 μL of 2,4-dinitrophenylhydrazine (DNPH) was added and the mixture was incubated for 30 min at room temperature. Then, 500 μL of 10% (*w*/*v*) trichloroacetic acid solution was added to the incubation mixture and the mixture was incubated on ice for 15 min to precipitate the proteins. The samples were then centrifuged, the supernatant discarded and the precipitate washed with 1 mL of ethyl acetate. This procedure was repeated 3 times. After washing, the samples were resuspended in 500 μL guanidine hydrochloride solution (6 M). The absorbance was measured at 370 nm. The antioxidant capacity was expressed in relation to the positive control according to formula (4):

(4) $$Antioxidant\;capacity\;\left(\%\right)=\frac{{Absorbance}_{positive\;control}-{Absorbance}_{sample}}{{Absorbance}_{positive\;control}}\times 100$$

#### 3.4.7. Determination of Antioxidant Effect on Model DNA Macromolecule

The double-stranded plasmid phiX RF1 DNA, with a length of 5386 base pairs and a molecular weight of 3.5 × 10^6^ Da, isolated from *E. coli*, was used to test the antioxidant effect of hydroxyl radicals in ground ivy extract generated by hydrogen peroxide and UV light on DNA. Prior to electrophoresis, a 1% (*w*/*v*) agarose gel was prepared using TAE buffer. The reaction mixture contained 0.1 μg mL^−1^ of plasmid, 0.1% (*v*/*v*) hydrogen peroxide solution and various concentrations of ground ivy extract (0.0125, 0.025, 0.075 and 0.25 mg mL^−1^) set to a final volume of 30 μL. All samples were subjected to UV irradiation for 16 min. The first negative control contained 0.1 μg mL^−1^ plasmid, whereas the second negative control contained the same concentration of plasmid but was exposed to UV radiation for 16 min. The third negative control contained the same concentration of plasmid but also contained a 0.1% (*v*/*v*) hydrogen peroxide solution. The positive control contained 0.1 μg mL^−1^ plasmid and 0.1% (*v*/*v*) hydrogen peroxide solution and was exposed to UV radiation for 16 min. After UV irradiation, 3 μL of stained loading buffer containing bromothymol blue (0.2 g), glycerol (50% (*v*/*v*) solution, 6 mL) and distilled water (4 mL) was added. Electrophoresis was performed for 1 h at a current of 150 mA. After electrophoresis, the gel was stained with ethidium bromide solution at a concentration of 20 μg mL^−1^ for 15–20 min. Bands were visualized using UV light, photographed and analyzed using the GelAnalyzer 19.1 software (www.gelanalyzer.com; by Lazar, I., Jr. and Lazar, I., Sr.; accessed on 20 September 2023). The original gel electrophoresis image (obtained gel stained with ethidium bromide and irradiated with UV radiation) can be found in the Supplementary Materials (Figure S1).

#### 3.4.8. Determination of Genotoxicity Using Alkaline Comet Assay

The comet assay was performed under alkaline conditions as described elsewhere [32], with some modifications. Cal27, HepG2, AGS and Caco-2 cell suspensions (5 mL each) were inoculated in Petri dishes at an initial concentration of 10^5^ cells mL^−1^. After attachment, the cells were treated with 0.0125, 0.025, 0.075 and 0.25 mg mL^−1^ concentrations of ground ivy extract. The negative control contained an appropriate culture medium. The treatment of cells lasted for 2 h or 24 h. After the treatment, the media containing the extract were removed from the Petri dishes and the cells were washed with PBS buffer, scraped and transferred into Eppendorf tubes. The cells were then centrifuged (5000 rpm, 5 min) and the supernatant was discarded. Cells were mixed with 100 μL of 0.5% (*w*/*v*) low melting point (LMP) agarose and smeared on previously prepared slides. After polymerization, the slides were coated with another layer of LMP agarose. After lysis, electrophoresis was performed at a current of 300 mA and a voltage of 25 V for 20 min. After electrophoresis, samples were stained with ethidium bromide solution (10 mg mL^−1^). Measurements were performed using an epifluorescence microscope (Leica Microsystems GmbH, Wien, Austria) with an excitation filter adjusted to 515–560 nm, while the Comet Assay II computer software was used for image analysis. A total of 100 comets per sample were scored. The parameters of tail intensity and tail moment were used as indicators of DNA damage.

#### 3.4.9. Statistical Analysis

All experiments were repeated at least 3 times, and each concentration was tested in triplicate. The obtained results were analyzed using Statistica software (v.14, TIBCO Software Inc., Palo Alto, CA, USA) using one-way ANOVA and Tukey post-hoc test. The results of the comet assay were analyzed using the Kruskal–Wallis test (between different extract concentrations at the same treatment time) and the Mann–Whitney U test (between 2 h and 24 h treatments at the same extract concentration). The selected significance level (*p*-value) was 0.05.

## 4. Conclusions

The cytotoxicity of ground ivy extract was found to be dependent on the type of cell line. Significant cytotoxic effects were observed only in the HepG2 cell line at an extract concentration of 0.25 mg mL^−1^ after 2 h of treatment and at an extract concentration of 0.0125 mg mL^−1^ after 24 h of treatment. A proliferative effect was observed following the treatment of AGS cell lines in 0.075 mg mL^−1^ of the extract. Further, the increase in extract concentration was followed by an increase in antioxidant effect on cellular model macromolecules, bovine serum albumin and plasmid phiX174 RF1 DNA. The extract exhibited genotoxic activity in Cal27 and HepG2 cell lines at a concentration of 0.075 mg mL^−1^, but this activity decreased with increasing duration of treatment. The extract also showed significant antimicrobial activity against the pathogenic bacteria *S. aureus* and *E. coli*.

## Figures and Tables

**Table 1:** Content (µg mg^−1^) of individual phenolic compounds in ground ivy extract.

Caffeic Acid	Chlorogenic Acid	Cryptochlorogenic Acid	Rosmarinic Acid	Rutin
1.59 ± 0.09	3.47 ± 0.03	1.16 ± 0.05	14.01 ± 0.17	5.01 ± 0.03

## Data Availability

Data are contained within the article and supplementary material.

## Supplementary Materials

The supporting information can be downloaded at: https://www.mdpi.com/article/10.3390/ijms242316975/s1.
