# Supplementary material for: Studying the Functional Potential of Ground Ivy (Glechoma hederacea L.) Extract Using an In Vitro Methodology
Source: Int J Mol Sci. 2023 Nov 30;24(23):16975. doi: 10.3390/ijms242316975 (PMC10707382; doi:10.3390/ijms242316975)
Supplement: Supplementary file 1 [file ijms-24-16975-s001.zip › ijms-2693602-supplementary.pdf]

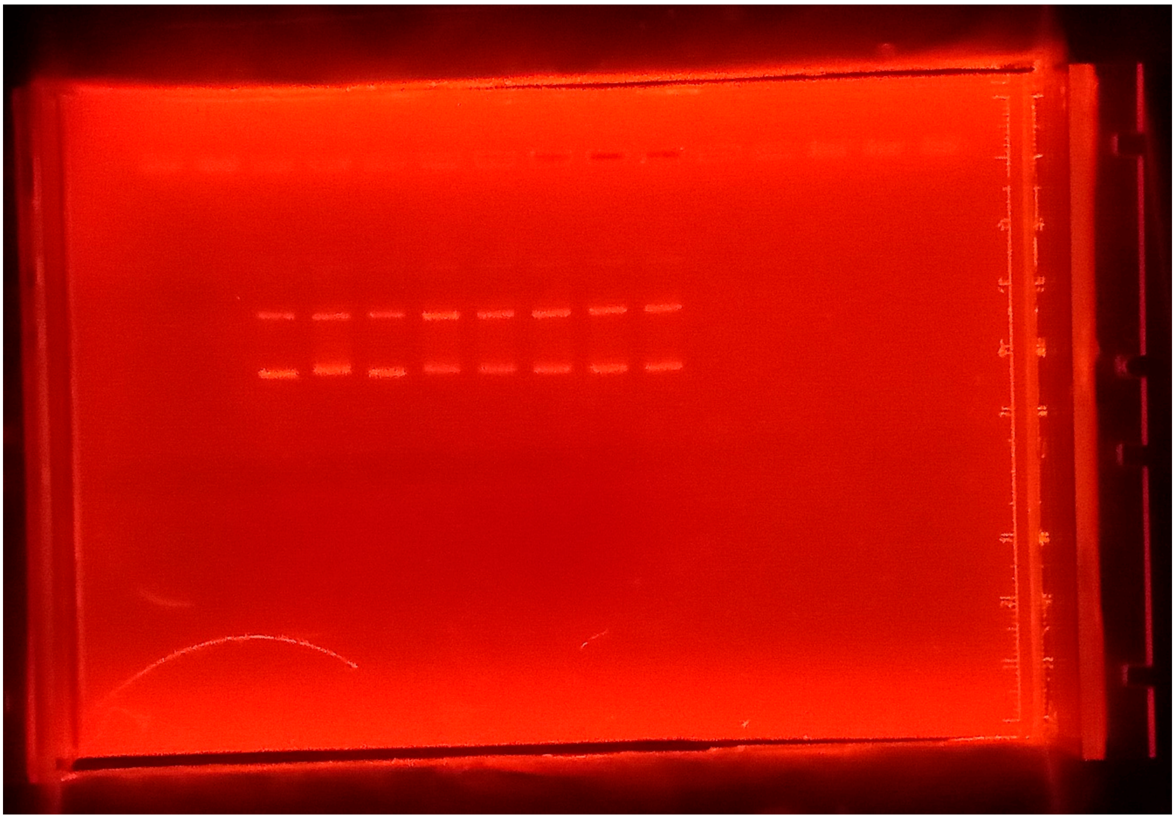

**Figure S1.** Full original image of gel electrophoresis - obtained gel stained in ethidium bromide and irradiated with UV radiation
